# Should the vent hole of posterior implant crowns be placed on the lateral surface? An *in vitro* study of the hydrodynamic feature of cement extrusion and retention ability

**DOI:** 10.1371/journal.pone.0276198

**Published:** 2022-10-20

**Authors:** Sixian Ye, Huangjun Zhou, Xingyu Lyu, Hao Feng, Min Liu, Cai Wen

**Affiliations:** 1 Department of Oral Implantology, The Affiliated Stomatological Hospital of Southwest Medical University, Luzhou, Sichuan, China; 2 Luzhou Key Laboratory of Oral & Maxillofacial Reconstruction and Regeneration, The Affiliated Stomatological Hospital of Southwest Medical University, Luzhou, Sichuan, China; 3 School of Stomatology, Southwest Medical University, Luzhou, Sichuan, China; 4 Department of Oral and Maxillofacial Surgery, The Affiliated Stomatological Hospital of Southwest Medical University, Luzhou, Sichuan, China; 5 Department of Oral Prosthodontics, The Affiliated Stomatological Hospital of Southwest Medical University, Luzhou, Sichuan, China; 6 Department of VIP Dental Service, The Affiliated Stomatological Hospital of Southwest Medical University, Luzhou, Sichuan, China; Kuwait University, Faculty of Dentistry, KUWAIT

## Abstract

Although placing a vent hole on the occlusal surface of the implant crown can reduce cervical marginal cement extrusion, it has disadvantages. Transferring the hole to the buccal or lingual surface of the posterior implant crown could therefore be an alternative solution. This study investigated the effect of transferring the vent hole to the lateral side of the implant posterior crown on the hydrodynamics of excess cement extrusion and the crown’s retention ability. Specially fabricated posterior implant crowns were divided into five groups: crowns with an occlusal hole (OH), occlusal lateral hole (OLH), middle lateral hole (MLH), cervical lateral hole (CLH), and no hole (NH). Each set of implant analog-abutment-crown specimens was wrapped in a polymethylacrylate base. The base of the implant crown was divided into four 90-degree quadrants along the diagonal of the square base with a pen mark. Cement was used to bond the crowns and the abutments, and the weight of cement extrusions at the vent holes and the abutment cervical margins were calculated. The distribution of cement extrusion at the margin was photographed in each quadrant, and the areas of surface coverage of cement extrusion were compared with ImageJ software. Retentive strength was measured as the dislocation force using a universal testing machine. One-way analysis of variance was used for result analysis. The cervical marginal cement extrusions of crowns with lateral holes (OLH, MLH, and CLH) were significantly less than that of NH crowns (P<0.05), but more than that of OH crowns (P<0.05). Subgroup analysis among the lateral hole groups indicated that the higher the position of the lateral hole, the lower the weight of the cement extrusion, and the smaller the total distribution area of cement extrusion. The cement extrusion distribution area was larger in the quadrant with the hole than in those opposite and next to the hole. Retention strength comparison indicated no significant difference between crowns with NH, OH, or lateral holes. Transferring the vent hole of the posterior implant crown to the lateral side could reduce cement extrusion at the cervical margin while reducing retention strength deterioration and the esthetic drawbacks caused by occlusal hole opening.

## Introduction

The high success rate of oral implants makes implant-supported prosthesis one of the most predictable options for patients with single or multiple tooth loss [1–4]. Implant crowns can be retained with either a screw or cement. Each retention type has its advantages and disadvantages, and data has shown either no statistical difference between screw- and cement-retained implant crowns [5–7], or a small difference between the mean values, which may not show clinical significance [8].

The advantages of cement-retained prosthesis include a simpler operating procedure, easier passive fit, better esthetic results, and lower prosthetic costs than screw-retained prosthesis [6, 9–12]. However, cement-retained prostheses are associated with several complications: cement residue at the gingival sulci around the abutment, especially underneath the gingival margin, is difficult to completely clean and can lead to peri-implant gingivitis, peri-implant inflammation, and ultimately implant failure [13–15]. Techniques have been proposed to reduce the cement residue underneath the gingival margin. It has been suggested that the placement of a vent hole on the implant crown could reduce the cement extrusion at the abutment margin without requiring more procedures or expenses. Compared with larger holes of around 2.5 mm in diameter, smaller holes around 1 mm in diameter have a similar performance in reducing the excess cement extrusion at the abutment cervical margin and cause little or no reduction in the retention ability and biomechanical strength of the crown [16, 17].

However, the occlusal holes on the implant crown, even the 1-mm vent holes, have drawbacks: 1) recurrent loss of the filled resin through the occlusal surface cannot be avoided; 2) aging of the filled resin results in esthetic flaws when the mouth is widely opened; and 3) a hole at the position of the screw access hole (SAH) leads to easier leakage of bacteria through the abutment SAH. To overcome these issues, the idea of transferring the hole to the buccal or lingual side of the crown can be considered to fulfill the goal of alleviating cement extrusion at the cervical margin and achieving superior esthetic results.

To the best of our knowledge, *in vitro* or clinical studies on implant posterior crowns with buccal/lingual vent holes which were not consistent with the SAH have not yet been reported. Therefore, this study aimed to investigate posterior cement-retained implant crowns with mini-sized vent holes placed on the lateral (buccal or lingual) side of the crowns and compare their influence on cement extrusion prevention and retention ability deterioration. The effect of placing the lateral hole at different heights (occlusal, middle, and cervical) of the crown was also discussed.

## Materials and methods

Dental implant analogs DAN38 (Dentium, Korea) were connected to titanium abutments and fastened at a torque of 30 Ncm as per the manufacturer’s recommendations. The abutments did not undergo grinding during the manufacturing of the crown, and the zirconia-made implant crowns in this experiment were designed by the same dental laboratory technician and manufactured by computer-aided design using 3Shape software (3Shape, Denmark) with the same morphology data, such that the only variation was the vent hole design. The occlusal surface of the crown was designed to be flat to facilitate standard weight pressuring in the cement extrusion experiment, and two traction holes were designed at the upper position of the crown for tensile traction in the retention strength experiment. All the vent holes were of mini sizes, approximately 1 mm in diameter. As the crown was flat on top, the buccal and lingual cusps of the tooth could not be reproduced on the crown. Since this study did not aim to compare the effect of the holes on the exact lingual or buccal side, the crowns were designed to be symmetrical in the bucco-lingual direction, so the buccal and lingual sides are collectively referred to as the lateral sides in this article.

**Fig 1:**
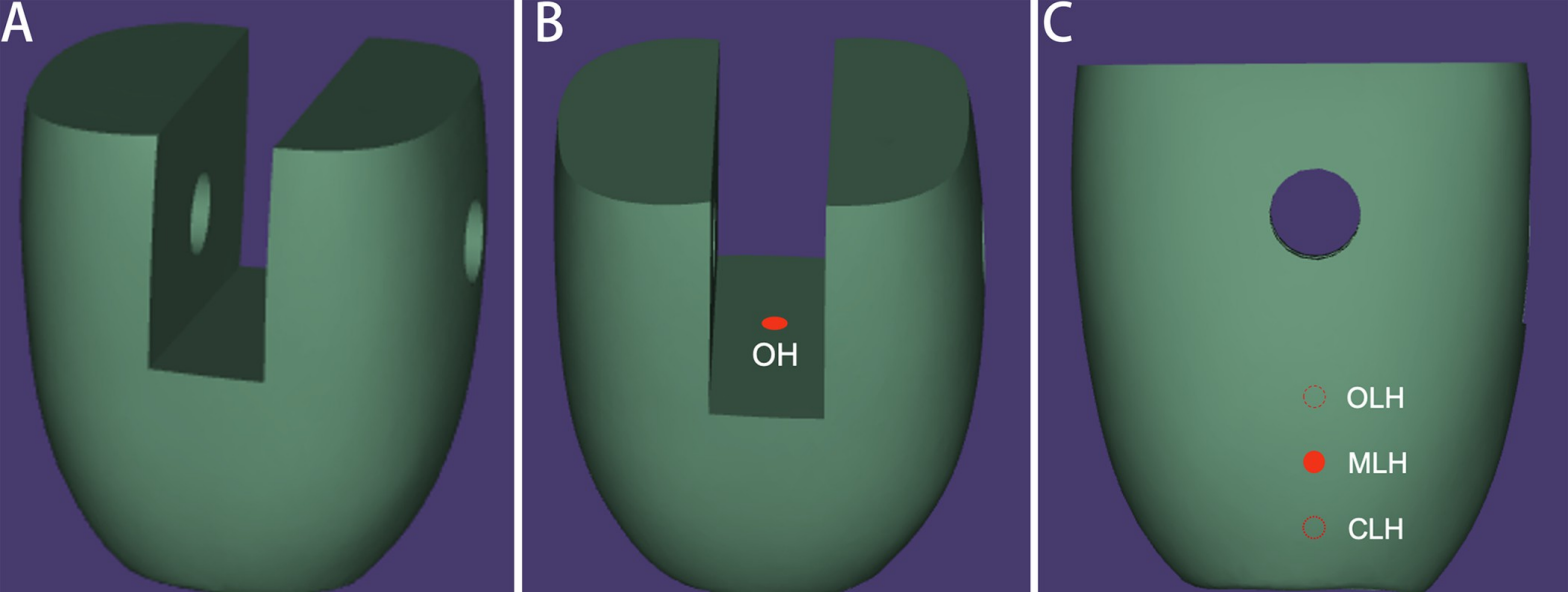
Specially fabricated implant crowns with different hole designs (A) lateral view of the specially fabricated NH crown, (B) crown with an occlusal vent hole (OH), (C) schematic diagram of the vent hole positions of the OLH, MLH, and CLH groups.

The crowns were divided into five groups according to the design and location of the holes: Group NH: crowns with no hole; Group OH: crowns with an occlusal vent hole; Group OLH: crowns with an occlusal height lateral vent hole; Group MLH: crowns with a middle height lateral vent hole; and group CLH: crowns with a cervical height lateral vent hole (Fig 1). There were three samples for each group, with the same morphology and hole designs.

**Fig 2:**
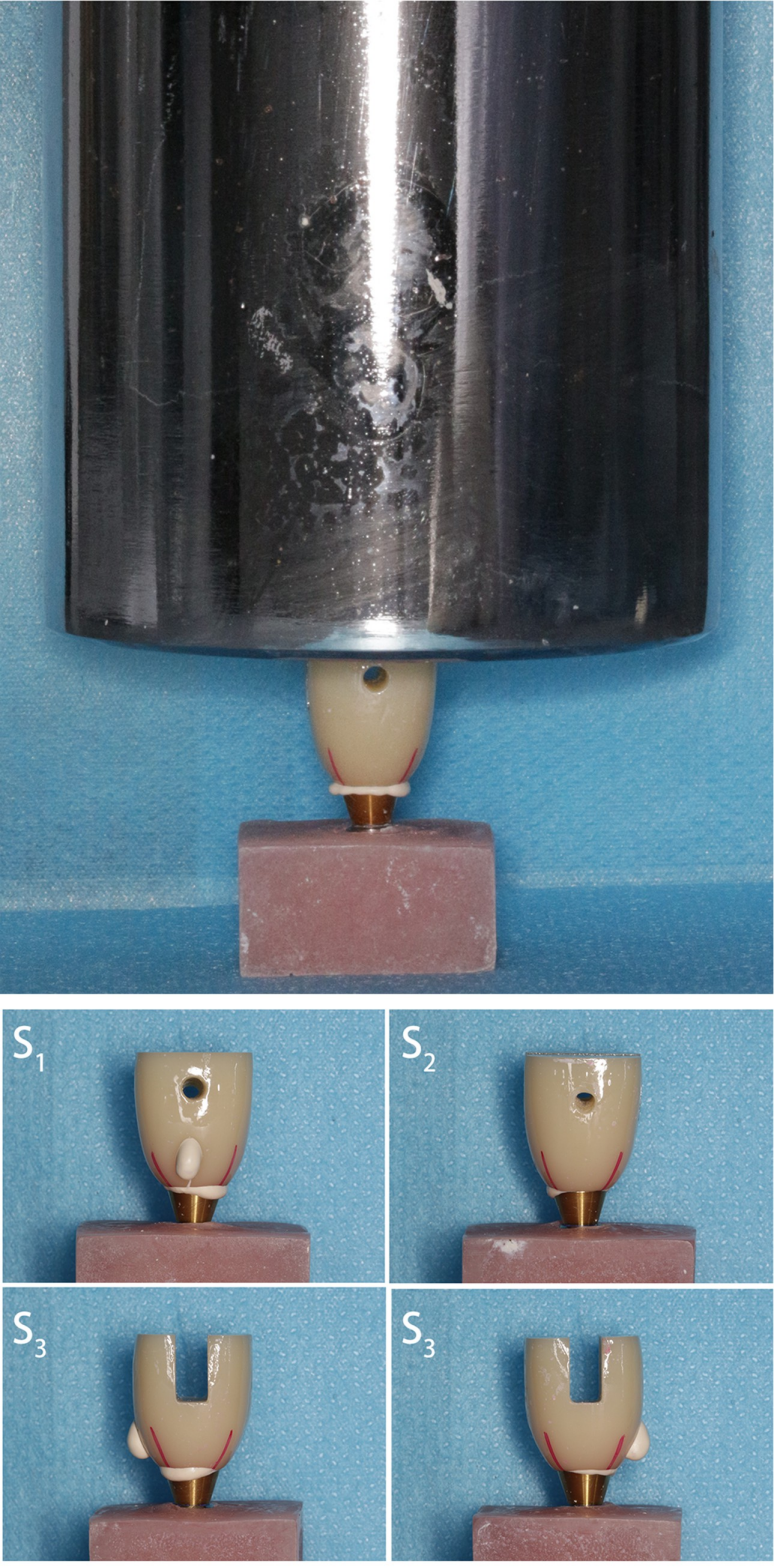
(A) Standardized weight pressuring after cement bonding. (B) Division of the quadrants according to the position of the hole. The quadrant with the lateral hole as S1, opposite the hole as S2, and the two quadrants adjacent to the hole as S3.

The analog-abutment-crown samples were assembled and embedded in a square-shaped base by self-curing polymethyl methacrylate (PMMA) resin, with the tangent directions of the lateral (buccal/lingual) sides of the crown parallel to the edge of the base. The base and the assembled crown were divided into four 90-degree quadrants along the diagonal of the square base, and a marker pen was used to identify the boundary of these quadrants on the abutments and analogs (Fig 2).

TempoCem NE Handmix cement (DMG, Germany) was used to bond the tooth crown and evaluate cement extrusion. According to the result of the pilot experiment, the loading amount of cement was standardized to around 40 mg each time. The base and accelerator of the cement were mixed by one experimenter (S.Y.). The cement was evenly coated inside the crown. The cement mixture and loading procedure was standardized to take 20–25 seconds altogether and it was ensured that the implant crown was passively seated on the abutment. A 2-kg-iron weight was then applied on top of the crown, and the pressure was maintained for 5 minutes.

### Weight of cement extrusion at the vent hole

After the cement solidified completely, the entire sample was weighed by an electronic scale (Mettler Toledo, China), and the pre-cement-removal weight was recorded as “m1.” A dental probe was used to thoroughly remove the excess cement flush with the crown surface from the vent hole in groups OH, OLH, MLH, and CLH. Thereafter, the sample was measured again, and the post-cement-removal weight of the sample was recorded as “m2”. The amount of cement extrusion at the vent hole was calculated as ΔM1 = m1-m2.

### Cement distribution at the abutment margin

**Fig 3:**
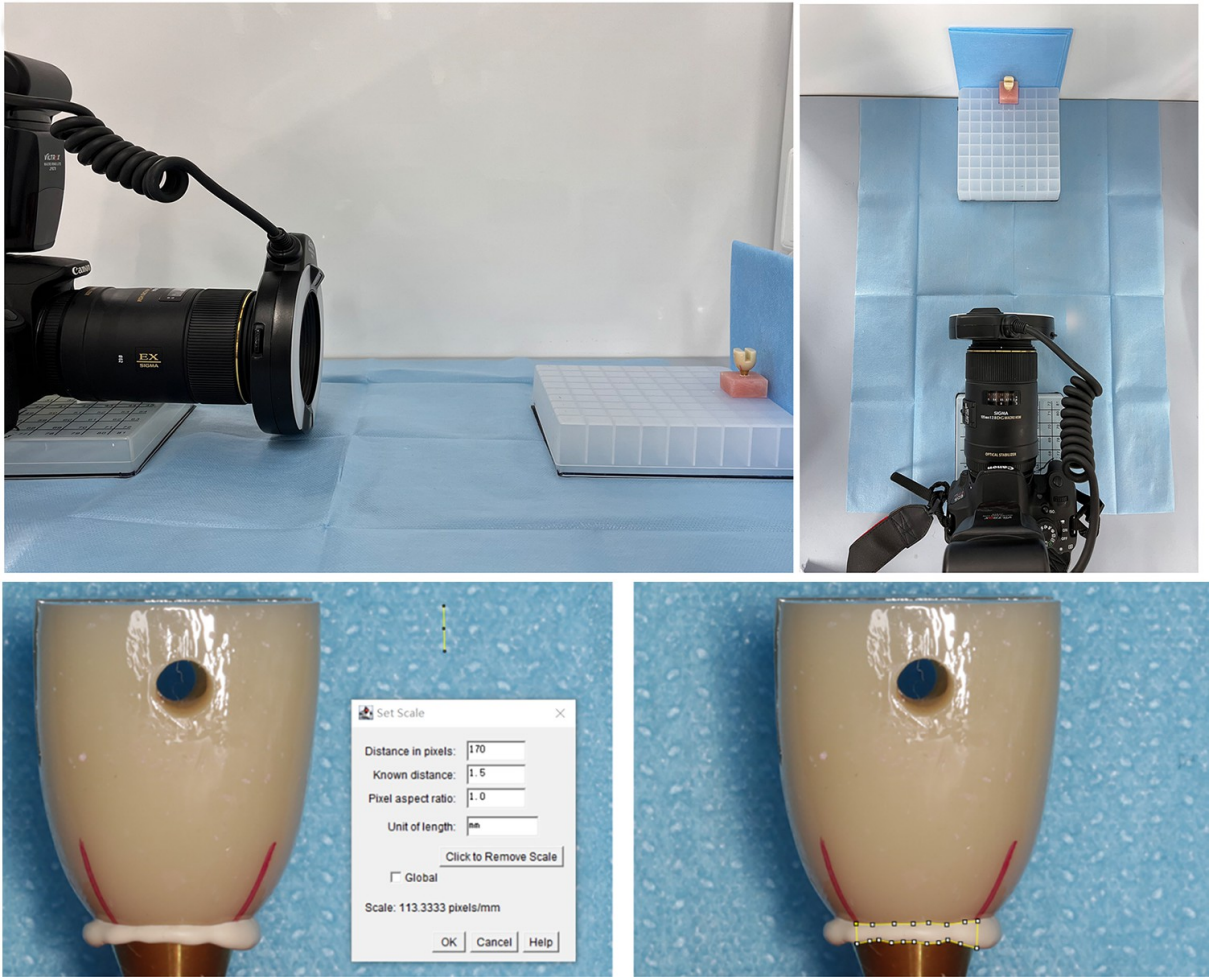
(A) Photographing the cemented crowns at a set photo studio and (B) ImageJ evaluation process for the calibration and calculation of the distributed cement extrusion areas.

The base-analog-abutment-crown system was fixed onto a set of a photo studio we made ourselves. A Canon single-lens reflex camera with a Sigma 105-mm prime lens was fixed, and the cement extrusion at the abutment margin was photographed with a magnification of 2.8X, an aperture of F20, and a shutter speed of 1/100 s. Only one quadrant of the crown was photographed in each picture, and a total of four pictures were taken per sample, including each side of the crown. The image processing software ImageJ (National Institute of Health, USA) was used to analyze the images from each group. After calibration, the distributed region of the extruded cement in the corresponding quadrant was chosen, and the area of cement was calculated by the software (Fig 3). The area obtained from the quadrant of the hole was recorded as S1, the quadrant opposite the hole as S2, and the area from the two quadrants, which were adjacent to S1 and S2, as S3. The total area of cement distribution from the four quadrants of each crown was summed together as the total cement extrusion area, S.

### Weight of cement extrusion at the abutment margin

After photographing, the extruded cement at the abutment margin was thoroughly removed by a probe, and gauze was used to scrub the crown and abutment margins. Thereafter, the sample was measured again, and the post-edge-cement-removal weight of the sample was recorded as “m3.” The amount of cement extrusion at the abutment margin was calculated using the formula ΔM2 = m2-m3.

### Retention strength test

**Fig 4:**
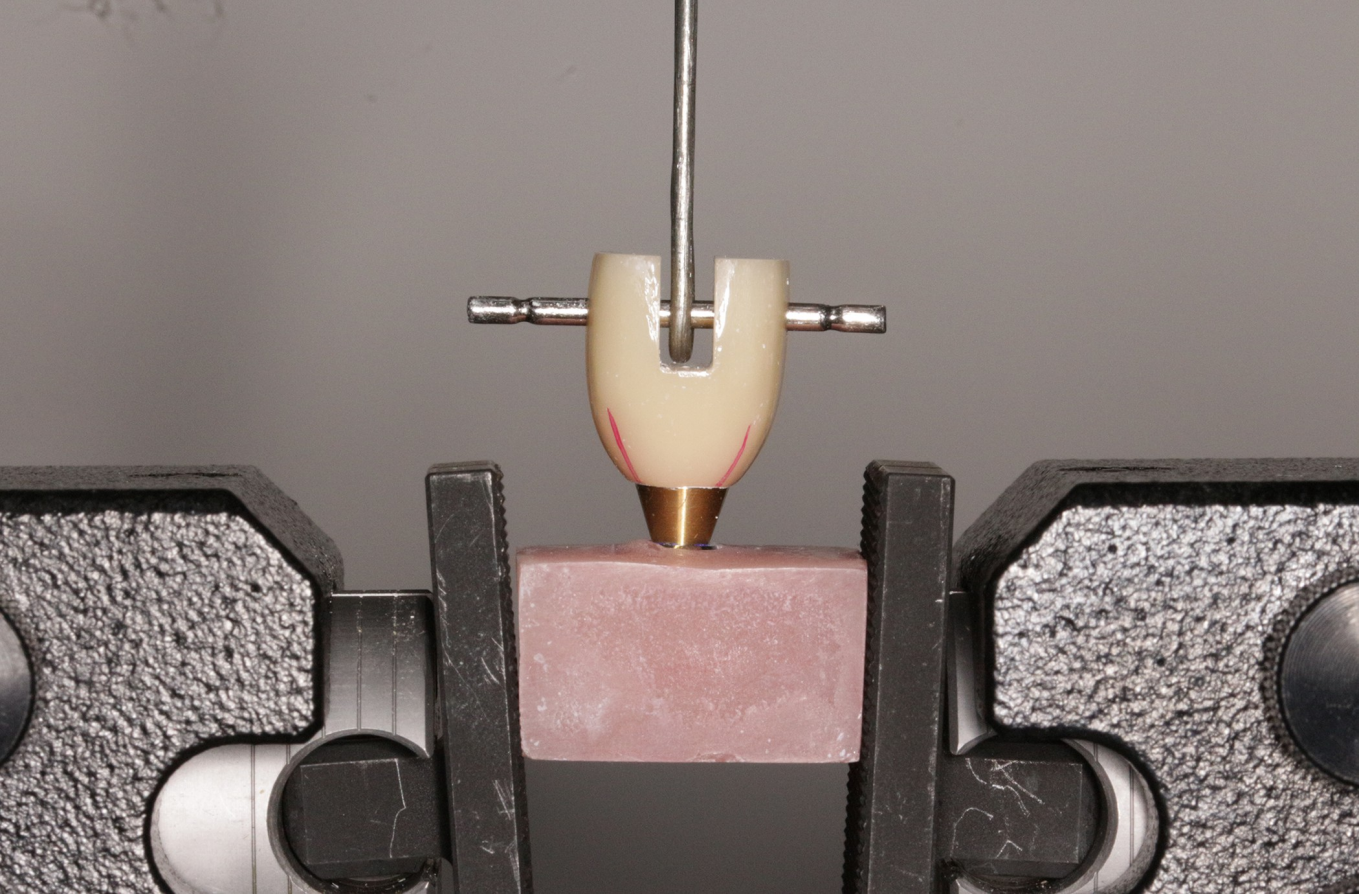
Retentive strength testing of the cemented implant crowns using a universal testing machine.

The samples were placed in the universal testing machine (Instron, USA), and an iron wire loop was passed through the holes of the crown. Tensile force was applied parallel to the longitudinal axis of the tooth at a speed of 1 mm/min until the crown was separated from the abutment. The stress-dislocation curve of the crown was recorded, and the highest point of the curve was considered the dislocation force as well as the retention strength of the crown (Fig 4).

After one set of experiments, large chunks of cement on the abutment and crown were removed, and the abutment and crown were soaked in 98% alcohol for 10 minutes and then cleaned with an ultrasonic vibration cleaner (Shumei KQ5200E, China) for 10 minutes. After the cement residue had been completely removed, the crowns were dried with clean air. Before each experiment, a six-sided sieve was used for the selection of the test sample out of the three samples in one group, and every experiment was conducted 10 times for each crown group.

Microsoft Excel (Microsoft Corp., USA) was used to record the data. Statistical Package for the Social Sciences software, SPSS 23.0 (IBM, USA) was used for data analysis, and Graphpad Prism 9 (Graphpad, USA) was used for figure drawing. The mean and standard deviation of the weights and distribution areas of cement extrusion at the vent hole and the abutment cervical margin were calculated, and the crown’s retention strength values were compared. Levene’s test was used to test for variance homogeneity. One-way ANOVA was used to analyze differences between groups if the data variance was homogeneous, while Welch’s ANOVA was applied if the variance was unequal, and pairwise comparisons for subgroups were performed. A p-value less than 0.05 was considered statistically significant.

## Results

For each group, three sets of analog-abutment-crown samples were fabricated. The sample size of the experiment was calculated with PASS15.0 software (NCSS, USA) based on the results of a pilot experiment (S1 Raw data), and the sample size of each group needed to be more than nine. The experiment was repeated 10 times out of 3 samples in each group with the help of a six-sided sieve.

### Cement extrusion at the vent hole

**Fig 5:**
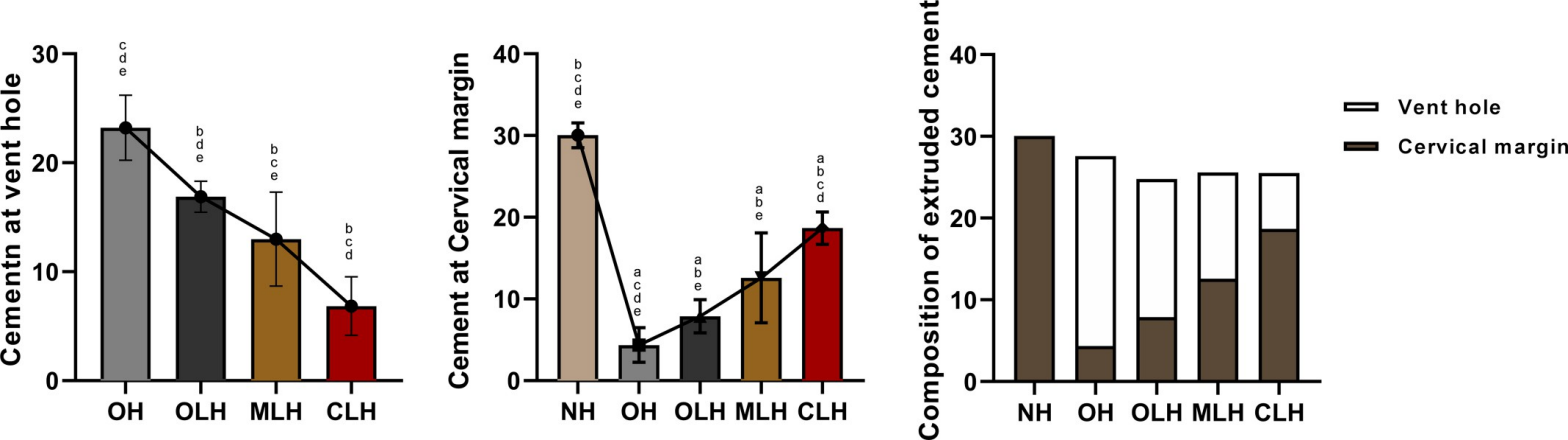
The cement extrusion at the (A) vent hole, (B) abutment cervical margin, and the (C) composition of the extruded cement from different sources. Lowercase superscript letters denote significant differences when comparisons were conducted among (a) NH, (b) OH, (c) OLH, (d) MLH, and (e) CLH (P< 0.05).

The weight of cement extrusion at the vent hole in groups OH (23.22±2.99 mg), OLH (16.89±1.43 mg), MLH (12.99±4.31 mg), and CLH (6.85±2.69 mg) decreased successively. Levene’s test showed homogeneity of variance in each group (P = 0.644). There was a significant difference in the weight of cement extrusion at the different hole positions (F = 51.287, P<0.001). Multiple intergroup comparisons of Tukey HSD test results showed that the differences among groups were statistically significant (P<0.001) (Table 1 and Fig 5A).

**Table 1:** Cement extrusion (mg) at the vent hole and the abutment cervical margin in each group (Mean ± SD).

Group	Vent hole	Cervical margin	Total
NH	-	30.04±1.51^b,c,d,e^	30.04±1.51^c,d,e^
OH	23.22±2.99^c,d,e^	4.36±2.11^a,c,d,e^	27.58±3.15^c^
OLH	19.89±1.43^b,d,e^	7.89±2.04^a,b,e^	24.78±1.14^a,b^
MLH	12.99±4.31^b,c,e^	12.59±5.50^a,b,e^	25.58±2.05^a^
CLH	6.85±2.69^b,c,d^	18.67±1.97^a,b,c,d^	25.52±2.36^a^
	ANOVA	Welch’s ANOVA	ANOVA
MS	472.008	-	45.658
Df	3	4	4
F	51.287	299.443^#^	9.802
p	<0.001	<0.001	<0.001

Lowercase superscript letters denote significant difference detected when multiple comparisons were conducted among (a) NH, (b) OH, (c) OLH, (d) MLH, and (e) CLH (*P*< 0.05). Superscript # denotes Welch’s ANOVA.

### Cement extrusion at the abutment margin

The maximum cement extrusion at the abutment cervical margin was in group NH (30.64±1.51 mg), and the minimum cervical cement extrusion was in group OH (4.36±2.11 mg). Heterogeneity of variance was detected by Levene’s test (P = 0.010). Welch’s ANOVA showed statistically significant differences between groups (F = 299.443, P<0.001). Multiple intergroup comparisons using the Game–Howell test showed no significant difference in the weight of cervical cement extrusion between groups OLH and MLH (P = 0.150), while differences in other groups were statistically significant (P< 0.05) (Table 1 and Fig 5B).

### Total weight of cement extrusion

The total amount of the extruded cement indicated that crowns with lateral holes extruded less cement than crowns with no hole and those with an occlusal hole. The lower the position of the lateral vent hole, the higher the proportion of cement extrusion from the abutment margin was. One-way ANOVA indicated significant differences between groups, and multiple intergroup comparisons showed significant differences between crowns with lateral openings and those without holes (Table 1 and Fig 5C).

### Areas of cement extrusion at the cervical margin

Group NH had the greatest cervical cement extrusion area (14.51±0.98 mm^2^), while group OH had the lowest area (4.74±1.32 mm^2^). Levene’s test showed that the variance of the data was unequal (P = 0.013). Welch’s ANOVA showed significant differences between groups (F = 141.36, p< 0.001). Multi intergroup Games–Howell test indicated that there was no significant difference between groups OH and OLH (P = 0.076) and between groups OLH and MLH (P = 0.275), while intergroup differences between the other groups were significant (Table 2).

**Table 2:** Total areas of cervical marginal cement extrusion (mm^2^) evaluated by photography (Mean ± SD).

				95% Confidence Interval for Mean						
Group	Mean	Std. Dev	Std. Error	Lower Bound	Upper Bound	Min	Max	Df	F	P
NH	14.51	0.98	0.31	13.81	15.20	12.13	15.40	4	141.360^#^	<0.001
OH	4.74	1.32	0.42	3.80	5.69	3.42	6.80			
OLH	6.10	0.68	0.22	5.62	6.59	4.85	6.87			
MLH	7.35	1.73	0.55	6.11	8.58	5.42	10.05			
CLH	10.28	1.04	0.33	9.53	11.02	8.94	12.37			

# indicates Welch’s ANOVA.

### Distribution of extruded cement in each quadrant

**Fig 6:**
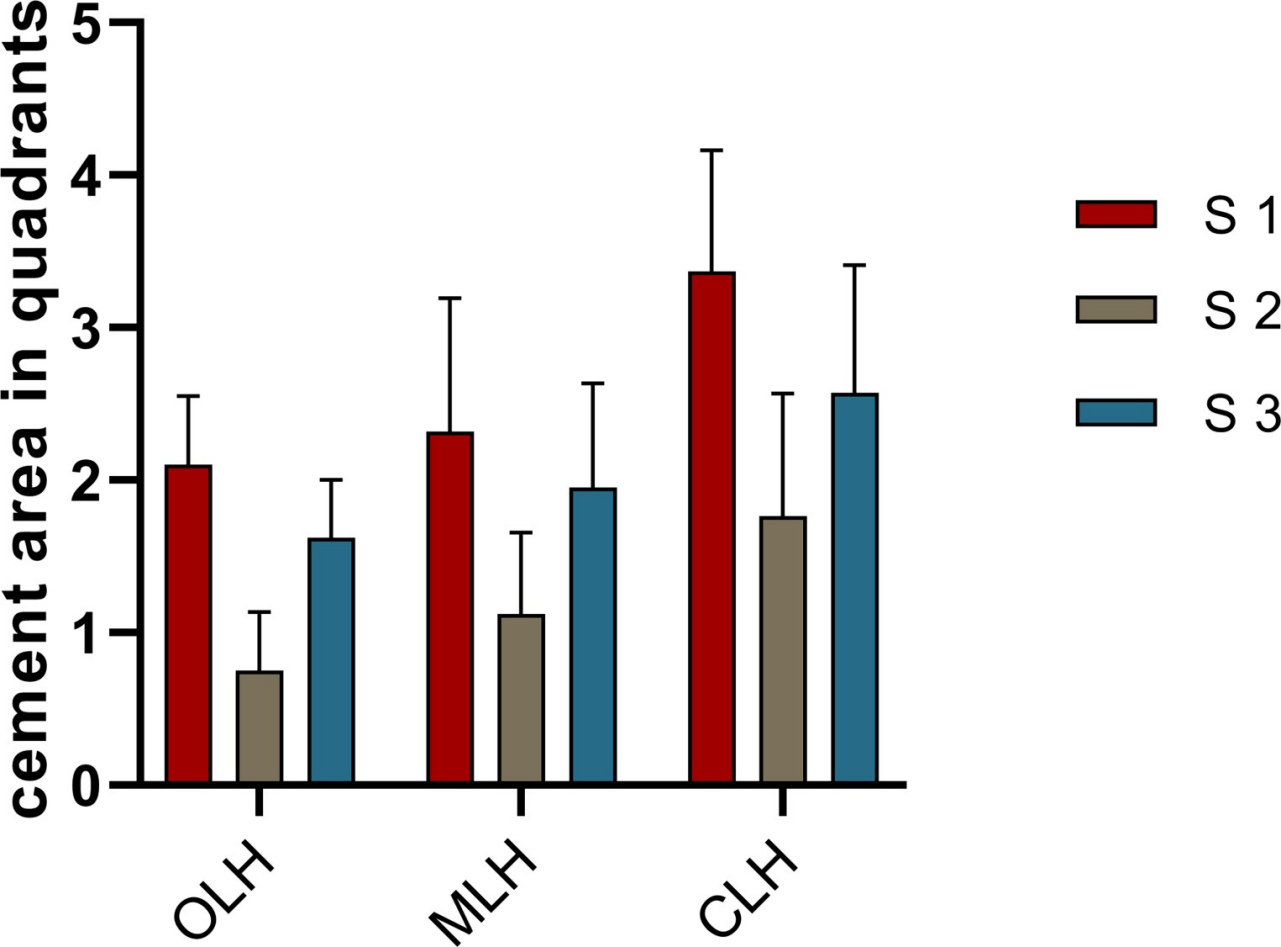
The mean cement extrusion areas for implant crowns with different lateral hole positions. (S1: quadrant of the hole, S2: quadrant opposite the hole, S3: quadrants adjacent to the quadrant of the hole).

For groups OLH, MLH, and CLH, the hydrodynamic feature of cement extrusion in each quadrant according to the position of the vent hole was compared. S2, the quadrant located opposite the vent hole, had the least cement extrusion, while S1, the quadrant of the vent hole, had the most cement extrusion when comparing groups OLH, MLH, and CLH. One-way ANOVA indicated significant differences among S1, S2, and S3 in the intragroup comparison (Table 3 and Fig 6).

**Table 3:** The areas of cervical marginal cement extrusion by quadrants for groups with a lateral vent hole (mm^2^).

	S1	S2	S3	MS	Df	F	P
OLH	2.10±0.45^cBC^	0.75±0.38^cAC^	1.62±0.38^cAB^	4.743	2	30.064	<0.001
MLH	2.32±0.88^cB^	1.12±0.53^AC^	1.95±0.69^cB^	3.831	2	7.715	0.002
CLH	3.37±0.79^abBC^	1.76±0.80^aAC^	2.57±0.84^abAB^	6.452	2	9.584	<0.001
MS	4.596	2.610	-	-	-	-	-
Df	2	2	2	-	-	-	-
F	8.631	7.300	10.835^#^	-	-	-	-
p	0.001	0.003	<0.001	-	-	-	-

Lowercase superscript letters denote significant differences detected when comparisons were conducted among (a) OLH, (b) MLH, and (c) CLH (P< 0.05); uppercase superscript letters denote significant differences when comparisons were conducted among (A) S1, (B) S2, and (C) S3 (P< 0.05). Superscript # denotes Welch’s ANOVA

### Retentive strength test

**Fig 7:**
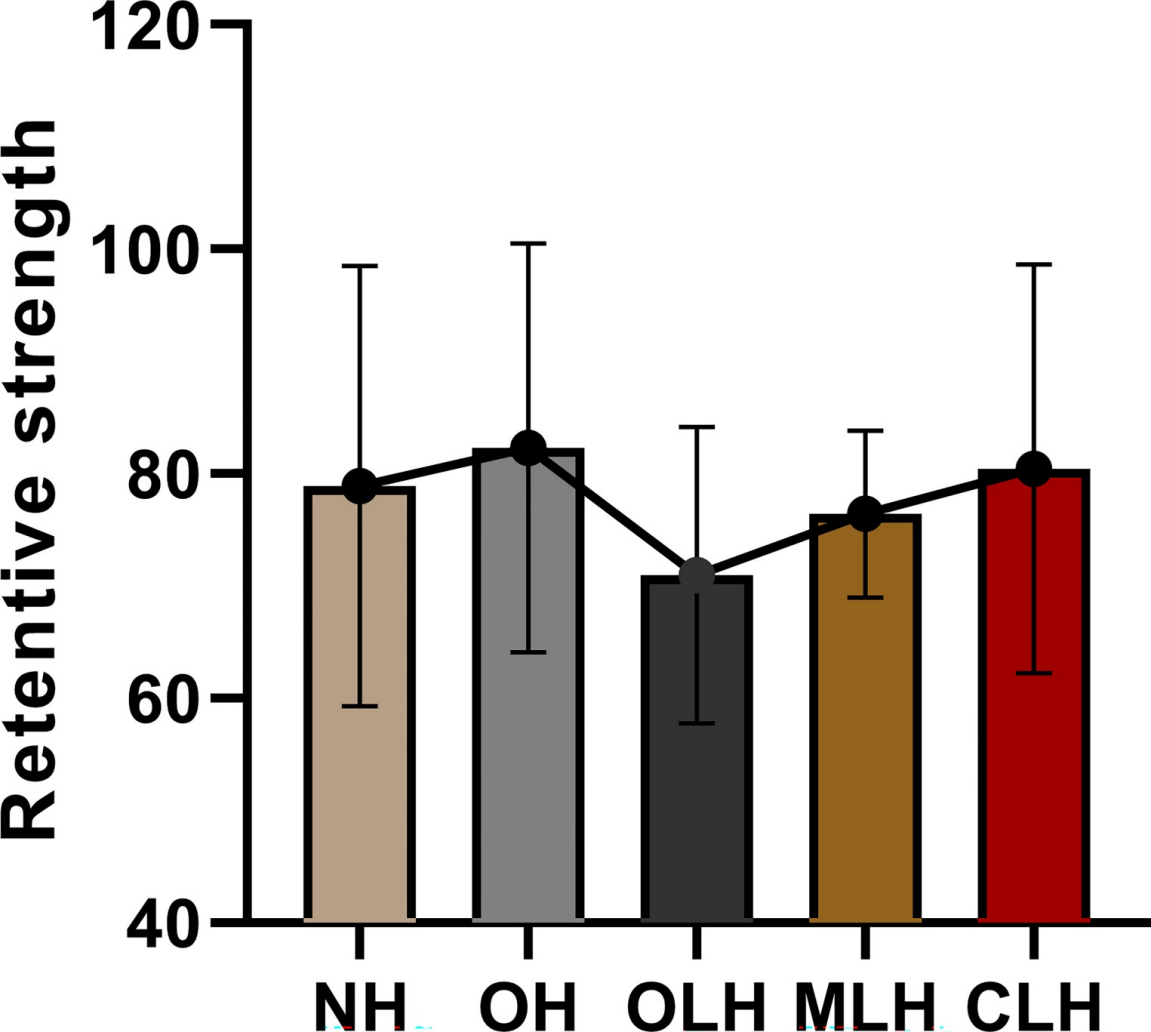
Retentive strength (N) for implant crowns with different hole designs.

Group OH had the highest retentive strength (82.32±6.21 N), while group OLH had the lowest retentive strength (70.95±4.17 N). Levene’s test showed homogeneity of variance (P = 0.161). ANOVA showed no significant difference in retentive strength values among groups (F = 0.759, P = 0.558) (Table 4 and Fig 7). For comparison of the lateral hole subgroups, the higher the position of the lateral hole, the weaker the crown retentive strength was.

**Table 4:** Retentive strength (N) of implant crowns with different hole designs (Mean ± SD).

				95% Confidence Interval for Mean							
Group	Mean	Std. Dev	Std. Error	Lower Bound	Upper Bound	Min	Max	MS	Df	F	P
NH	78.90	19.62	6.21	64.86	92.94	48.37	110.84	193.781	4	0.759	0.558
OH	82.32	18.21	5.76	69.29	95.35	49.55	107.29				
OLH	70.95	13.20	4.17	61.51	80.38	52.70	95.30				
MLH	76.41	7.45	2.36	71.08	81.74	62.86	85.54				
CLH	80.44	18.18	5.75	67.44	93.44	65.21	119.82				

## Discussion

The purpose of this study was to investigate the placement of a vent hole in a posterior implant crown on its lateral side (either buccal or lingual) and to evaluate its effect on the amount of cement extrusion and retention performance. To the best of the authors’ knowledge, this has not been discussed yet. According to the results of this study, a lateral vent hole can significantly decrease the cement extrusion at the abutment cervical margin compared with crowns lacking a hole, but it does not reduce cervical cement extrusion better than in crowns with an occlusal hole. There was no significant difference in the retention force between crowns with lateral vent holes and those without a hole.

There are two main types of implant support dental crowns: screw-retained and cement-retained ones. Screw-retained implant crowns can completely prevent peri-implant inflammation caused by cement residue, but their cost is higher, and mechanical complications, such as screw loosening or fractures, are unavoidable [6, 10]. Cement-retained crowns have the advantages of lower costs and easy passive fit between implant components. Although cement-retained implant crowns are favored by dentists for their low cost and easy handling, their disadvantage is obvious: it is difficult and sometimes impossible to avoid excessive subgingival cement residue, which can lead to peri-implant disease [11, 18]. Due to the deleterious effect of the extruded cement residue, attempts have been made to find methods to eliminate these drawbacks, such as opening holes of different diameters on the crown [19, 20], increasing the unfilled space of the abutment screw access hole [19], loading a precise amount of cement on the crown [21], placing the crown’s margin supragingivally [22], using polytetrafluoroethylene tape or a rubber dam as a barrier [23, 24], and applying customized abutment analogs prior to final cementing to obtain a customized cement film thickness [25]. Of all these methods, opening a vent hole on the crown is the easiest, and it requires no additional time or costs, making it practical and worthy of clinical application [26].

At present, different dentists have different practices when it comes to opening a hole on the crown. For the anterior teeth, the hole on the implant crown is usually on the lingual fossa, while for the posterior teeth, it is usually on the occlusal surface. The size of the hole is generally between 2.5 and 3 mm to allow a wrench to enter with ease. However, occlusal hole openings, especially the regular 2.5–3 mm hole, may cause recurrent shedding of restorative materials (e.g. composite resin) [27] and may cause deterioration of the fracture strength of the zirconia crown [16]. Nevertheless, a previous study showed that patients and dentists have different esthetic attitudes toward implant crowns with holes as well as different acceptability [18]. While dentists generally take holes on the implant crown for granted, most patients may not be aware that they would have holes on the implant crown, especially on the occlusal surface, when they begin treatment. Therefore, there should be a balance between pursuing better esthetic results of implant crowns and reduction of excess cement extrusion around the abutment cervical margin, with less possibility of recurring follow-up for complications related to the resin filling [28].

Studies have shown that vent holes with smaller diameters (1 mm) can also have advantages for cement extrusion reduction without affecting the retention ability compared with crowns having a regular larger hole (2.5 mm) [17]. Therefore, it is a novel attempt to change the location of the vent hole to the buccal or lingual side of the posterior crown since that design does not pose esthetic risks. As far as we know, the features of the buccal or lingual vent hole on a posterior implant crown have not been previously reported.

The findings of this study demonstrate the relationship between the amount of cement extrusion and the design of the hole. The amount and areas of cement extrusion at the abutment cervical margin of crowns with holes were lower than those of NH crowns. During the cementing process, air and cement inside the crown would be squeezed out. If no vent hole is present, excess cement can only squeeze out through the margin of the crown and may be extruded deep into the subgingival surrounding of the implant. Vents on the crown provide passage for the cement and air to be squeezed out, therefore, the hydrodynamic pressure at the abutment cervical margin would be reduced when the crown is seated.

Comparison between occlusal and lateral openings showed that as the position of the openings moves cervically, the amount of abutment cervical cement extrusion increases. A possible explanation for this is that during cementation, the cement is squeezed by the lower abutment, and the cement inside the upper interfacial space undergoes higher hydraulic liquid pressure, which makes the more occlusally positioned vent hole provide better relief for cement pressure and less cement extrusion at the abutment cervical margin. Therefore, it is suggested that the vent hole should be located more occlusally when placing it on the lateral side of a crown.

Crowns with lateral openings have special advantages. First, since the implant is recommended to be positioned corresponding to the functional cusp of the tooth, the occlusal hole of the crown may be present near the functional cusp and the filling resin at the vent hole would have to withstand the repeated pressure from mastication, which would increase the possibility of recurrent shedding of the filled resin. Placing the hole on the lateral surface of the crown would help move the hole away from areas of occlusal stress, reducing the risk of resin shedding, and thereby helping avoid frequent patient follow-up [27, 29]. Second, the resin materials in the mouth would have an aging effect coupled with food residues [30], which together would cause the discoloration of the resin [31]. Color differences between the resin and crown may cause esthetic problems when the mouth is wide open [32]. Conversely, holes on the lateral sides (whether buccal or lingual) could significantly reduce these esthetic concerns. Finally, the occlusal hole on the crown usually connects with the interior abutment SAH directly, which would become a channel for bacterial colonization by microleakage, long-term masticatory force, and the cold-hot cycle aging effect [33, 34]. Transferring the hole to the lateral side and away from the entrance of SAH may help minimize microleakage and bacterial colonization inwards.

As the samples had buccal-lingual symmetry, this study did not compare the superiority between buccal and lingual holes. The side of the hole opening should be comprehensively determined according to the actual mouth opening condition and the scope of the esthetic zone. A lingual opening hole may cause the sensation of a foreign body. Though negligible for most patients, the esthetic drawbacks of buccal openings can be bothersome. Generally speaking, it is better to open the hole on the lingual side to avoid the esthetic defect in the esthetic zone; however, in the molar zone, leaving the hole on either the buccal or lingual side is acceptable, and a buccal hole could help avoid the possible sensation of a foreign body.

For the cement extrusion distribution evaluation, in addition to the conventional comparison of cement extrusion weight, a new technique involving photograph records and a comparison of distributed cement areas in quadrants according to the hole position was utilized in this study. The cement distribution areas of crowns with holes were significantly lower than those of crowns without holes. The quadrant in which the hole was located had more cement extrusion compared with that in the other quadrants, and the higher the position of the lateral hole, the less the spillover area. This is also the first time the phenomenon that cervical cement extrusion increased in the quadrant of the opening is reported, which seems to be contrary to the expectation that there will be less cement extrusion in the quadrant where the opening is located, and the hydrodynamics of this phenomenon need to be further studied.

### Limitations

Although our implant crown samples are from the same computer data and were made by the same laboratory technician, there are inevitable individual differences between samples because of the fabrication process. The varying internal volume between the abutment and the crown would make experimental data unstable, leading to differences not quite resulting from the technician’s operation error, but rather, bias from the fabrication process. Also, the scrap rate of the crown’s fabrication and drilling is high. Therefore, though we had 10 sets of data for each group, we made 3 instead of 10 samples in each group for the sake of reducing experimental bias.

To make the experiment more repeatable, two-component temporary cement was used instead of permanent cement, such as resin cement or glass ionomer. The adhesive strength of permanent cement is too strong and so can either cause crown deformation or mask the subtle effect of the hole position on the retention force. We used mechanical removal and an ultrasonic vibration cleaner to thoroughly remove the cement before the next experiment.

## Conclusion

Within the limitations inherent to *in vitro* studies, the following conclusions were drawn:

1. The cervical excess cement extrusion was less for crowns with lateral holes than for NH crowns in terms of the weight and distribution area. The higher the position of the lateral hole, the lower the cement extrusion at the abutment margin.
2. Retention strength comparison indicated no significant differences among crowns with NH, OH, or lateral holes, and the position of the lateral holes does not affect its retention ability.

Transferring the vent hole of the cement-retained implant crown to its lateral surface can simultaneously eliminate the extruded cement at the abutment cervical margin, reduce the aesthetic defect associated with the occlusal hole, and eliminate recurrent shedding of resin; thus, it has potential for clinical application.

## Supporting information

S1 Fig The specially fabricated crowns used in the experiment. The specially fabricated crowns without hole, with occlusal hole or lateral hole on different height; and the damaged crowns during lateral hole drilling.
(DOCX)

S1 File The raw data of the experiment. It contain the raw data of the amount, area of cement extrusion in total or in quadrants,and the retentive strength of crowns.
(XLSX)

S2 File The sample size calculation. It contain the process of sample size calculation using software.
(DOCX)

S1 Raw data (XLSX)
