# Supplementary material for: Should the vent hole of posterior implant crowns be placed on the lateral surface? An in vitro study of the hydrodynamic feature of cement extrusion and retention ability
Source: PLoS One. 2022 Oct 20;17(10):e0276198. doi: 10.1371/journal.pone.0276198 (PMC9584542; doi:10.1371/journal.pone.0276198)

####
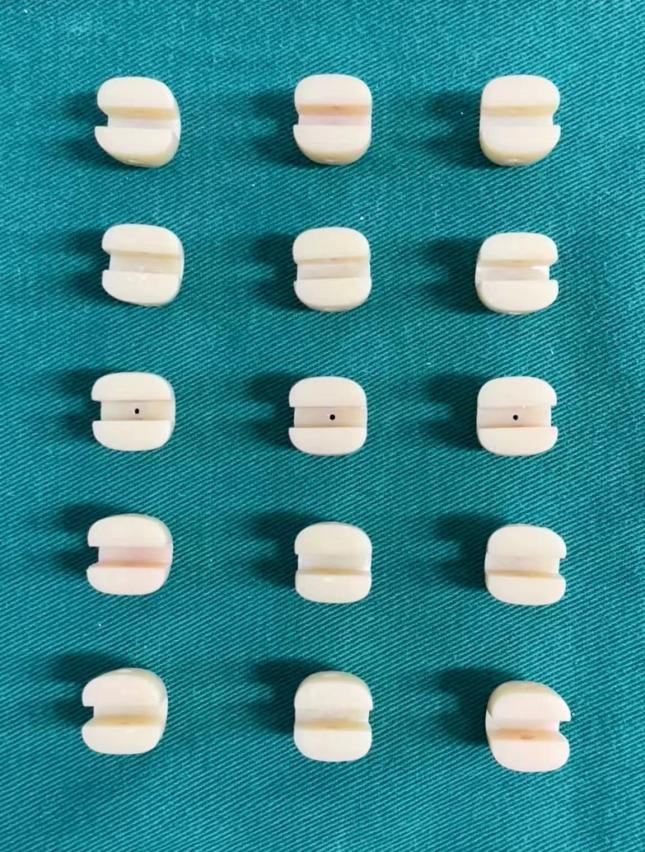
The specially fabricated crowns used in the experiment.

**The damaged crowns during lateral hole drilling in the experiment.**


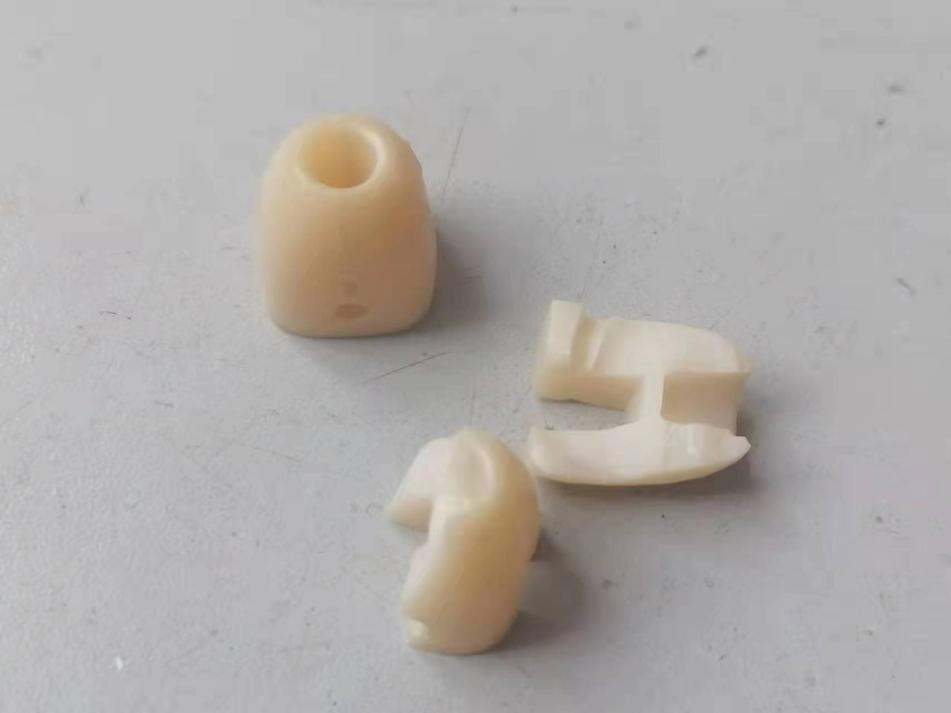

Supplement: S1 Fig — The specially fabricated crowns without hole, with occlusal hole or lateral hole on different height; and the damaged crowns during lateral hole drilling. (DOCX) [file pone.0276198.s001.docx]
