# Supplementary material for: Should the vent hole of posterior implant crowns be placed on the lateral surface? An in vitro study of the hydrodynamic feature of cement extrusion and retention ability
Source: PLoS One. 2022 Oct 20;17(10):e0276198. doi: 10.1371/journal.pone.0276198 (PMC9584542; doi:10.1371/journal.pone.0276198)
Supplement: S2 File — It contain the process of sample size calculation using software. (DOCX) [file pone.0276198.s003.docx]

**Sample Size Calculation**

The sample size of the experiment was calculated using the PASS15.0 software (NCSS, LLC, Utah, USA) before the experiment. The means (M) and standard deviations (SD) used in the calculation were obtained from a pilot experiment. The cervical marginal cement extrusion in these groups are 26.02±2.84 mg (NH); 6.01±1.92 mg (OH); 8.50±1.53 mg (OLH); 13.81 ± 4.62 mg (MLH); 17.31±4.86 mg (CLH). The analysis module "One-Way Analysis of Variance F-Tests" of the PASS with a statistical power of 90% was adopted. Meanwhile, the significance level was set to 5% and the number of groups was set to 5. The sample size of each group that required at least 3 samples was calculated (Table 1).

We then repeated the above steps and input the Retentive strength of pilot experiment into the PASS15.0 software, i.e., 76.76±11.18N (NH); 82.96±9.38N (OH); 71.14±11.26N (OLH); 78.99±8.93 N (MLH); 92.81±11.51N (CLH). The minimum sample size of each group was calculated as 9 (Table 2). The sample size of 10 in each group is not very large, but it exceeded the minimum sample size estimated by the PASS software and meets the statistical requirements.

Table 1

|  | Average  n |  |  |  | SD of Means | SD | Effect Size |  |
| --- | --- | --- | --- | --- | --- | --- | --- | --- |
| Power |  | G | N | K | σm | σ |  | Alpha |
| 1.0000 | 2 | 5 | 10 | 1.00 | 7.06 | 1.53 | 4.6136 | 0.05 |
| 1.0000 | 2 | 5 | 10 | 1.00 | 7.06 | 1.92 | 3.6765 | 0.05 |
| 0.9806 | 2 | 5 | 10 | 1.00 | 7.06 | 2.84 | 2.4855 | 0.05 |
| 0.9746 | 3 | 5 | 15 | 1.00 | 7.06 | 4.62 | 1.5279 | 0.05 |
| 0.9594 | 3 | 5 | 15 | 1.00 | 7.06 | 4.86 | 1.4524 | 0.05 |

Table 2

|  | Average  n |  |  |  | SD of Means | SD | Effect Size |  |
| --- | --- | --- | --- | --- | --- | --- | --- | --- |
| Power |  | G | N | K | σm | σ |  | Alpha |
| 0.9167 | 6 | 5 | 30 | 1.00 | 7.23 | 8.93 | 0.8098 | 0.05 |
| 0.9398 | 7 | 5 | 35 | 1.00 | 7.23 | 9.38 | 0.7709 | 0.05 |
| 0.9245 | 9 | 5 | 45 | 1.00 | 7.23 | 11.18 | 0.6468 | 0.05 |
| 0.9205 | 9 | 5 | 45 | 1.00 | 7.23 | 11.26 | 0.6422 | 0.05 |
| 0.9075 | 9 | 5 | 45 | 1.00 | 7.23 | 11.51 | 0.6283 | 0.05 |
